# A Score-Fusion Method Based on the Sine Cosine Algorithm for Enhanced Multimodal Biometric Authentication

**DOI:** 10.3390/s26010208

**Published:** 2025-12-28

**Authors:** Eslam Hamouda, Alaa S. Alaerjan, Ayman Mohamed Mostafa, Mayada Tarek

**Affiliations:** 1 Computer Science Department, Faculty of Computers & Information, Mansoura University, Mansoura 35516, Egypt; eslam_foad@mans.edu.eg (E.H.); mayada_tarek@mans.edu.eg (M.T.); 2 Computer Science Department, College of Computer and Information Sciences, Jouf University, Jouf 72388, Saudi Arabia; 3 Information Systems Department, Faculty of Computers and Informatics, Zagazig University, Zagazig 44519, Egypt; 4 Information Systems Department, College of Computer and Information Sciences, Jouf University, Jouf 72388, Saudi Arabia

**Keywords:** multimodal biometrics, metaheuristic-optimization, biometric fusion, Sine Cosine Algorithm (SCA)

## Abstract

Score fusion is a technique that combines the matching scores from multiple biometric modalities for an authentication system. Biometric modalities are unique physical or behavioral characteristics that can be used to identify individuals. Biometric authentication systems use these modalities to verify or identify individuals. Score fusion can improve the performance of biometric authentication systems by exploiting the complementary strengths of different modalities and reducing the impact of noise and outliers from individual modalities. This paper proposes a new score fusion method based on the Sine Cosine Algorithm (SCA). SCA is a meta-heuristic optimization algorithm used in various optimization problems. The proposed method extracts features from multiple biometric sources and then computes intra/inter scores for each modality. The proposed method then normalizes the scores for a given user using different biometric modalities. Then, the mean, maximum, minimum, median, summation, and Tanh are used to aggregate the scores from different biometric modalities. The role of the SCA is to find the optimal parameters to fuse the normalized scores. We evaluated our methods on the CASIA-V3-Internal iris dataset and the AT&T (ORL) face database. The proposed method outperforms existing optimization-based methods under identical experimental conditions and achieves an Equal Error Rate (EER) of 1.003% when fusing left iris, right iris, and face. This represents an improvement of up to 85.89% over unimodal baselines. These findings validate SCA’s effectiveness for adaptive score fusion in multimodal biometric systems.

## 1. Introduction

Multi-biometric systems are personal identification and verification systems that use multiple biometric traits to identify an individual. Biometric traits are unique physical or behavioral characteristics that can be used to identify a person, such as fingerprints, facial features, iris patterns, voice patterns, and gait. Multi-biometric systems are more accurate and secure than single-biometric systems because they use multiple traits to authenticate an individual. It can be used in various applications, including Access control, Law enforcement, Border Security, and Commercial applications. Multi-biometric systems are a rapidly evolving field of research and development, with new biometric modalities and fusion algorithms being introduced steadily [1].

Multi-biometric fusion systems can be classified into three main types: Feature-level fusion, Score-level fusion, and Decision-level fusion. In feature-level fusion, the features extracted from the different biometric traits are combined into a single feature vector. This feature vector is then used to train a classifier, which is used to authenticate the individual. Feature-level fusion enhances performance by synergizing complementary information from diverse biometric modalities [2]. In score-level fusion, the matching scores from the different biometric traits are combined to decide whether to authenticate the individual. Several score-level fusion algorithms can be used, such as weighted sum, majority voting, and Dempster-Shafer fusion [3]. Decision-level fusion combines decisions from multiple sources to improve accuracy. It is a widely used technique in machine learning, artificial intelligence, and signal processing. Common decision fusion algorithms include majority voting, weighted average, and Bayesian fusion [4]. Score-level fusion has many advantages over other multi-biometric fusion methods, including:

- Simplicity: Score-level fusion is relatively simple to implement in terms of the algorithms required and the amount of data needed. This makes it a good choice for applications where resources are limited.
- Efficiency: Score-level fusion is also very efficient, meaning that it can be implemented to run quickly on low-powered devices. This is important for applications where real-time performance is critical.
- Robustness: Score-level fusion is robust to noise and environmental variations, meaning it can still perform well even when the biometric data is of poor quality or the system operates in a challenging environment.
- Flexibility: Score-level fusion can combine the matching scores from any biometric modality, making it a very flexible fusion method.

### Problem Statement

Deep learning has been used to develop new score-level fusion methods for Multi-biometric systems [5]. These methods improve the accuracy and security of score-level fusion. However, its limitations include its complexity, data requirements, and need for interpretability. On the other hand, the performance of score-level fusion algorithms based on weighted sum is sensitive to the selection of fusion parameters; the weights assigned to the matching scores from different biometric modalities can significantly impact the system’s accuracy. It is important to carefully tune the fusion parameters for a specific Multi-biometric system to achieve optimal performance.

This study presents a score-level fusion algorithm for Multi-biometric authentication systems. It employs the Sine Cosine Algorithm (SCA) to enhance the Multi-biometric system’s recognition accuracy. The contributions for this study are summarized as follows:

1- Proposes a novel SCA-based adaptive score fusion framework for multimodal biometrics;
2- Demonstrates superior performance over PSO and GWO using various performance metrics.
3- Validates the approach on realistic iris-face combinations using CASIA and ORL datasets

The SCA is a meta-heuristic optimization algorithm used in various optimization problems [6]. The proposed method for multimodal biometric fusion first extracts features from multiple biometric sources, such as fingerprints, iris scans, and facial images. It then computes intra- and inter-class scores for each modality, which measure the similarity between biometric samples from the same individual and different individuals, respectively. The scores are then normalized for each user, and various aggregation techniques combine the scores from different modalities. An SCA optimization algorithm finds the optimal parameters for the aggregation process. Our method learns to combine the matching scores from different biometric modalities based on a training dataset of labeled biometric samples. This allows the algorithm to adapt to the biometric modalities’ specific characteristics. Additionally, our method is relatively easy to implement and can be used with various biometric modalities. It has the potential to be used in a wide range of applications, such as access control systems, law enforcement and security systems, financial and banking systems, healthcare systems, and mobile devices.

The remaining sections of this study are organized as follows: Section 2 summarizes the relevant research works for Multi-biometric systems. Section 3 describes the proposed score fusion algorithm. Section 4 introduces the experiments and performance evaluation. Finally, the paper is concluded in Section 5.

## 2. Related Works

Biometric fusion combines multiple biometric modalities to improve the accuracy, robustness, and cost of biometric systems. It works by combining information from multiple modalities to compensate for the weaknesses of each modality. The recent methods for Multi-biometric fusion are discussed in the following subsections.

### 2.1. Feature-Level Fusion

Feature-level fusion is often used in applications with noisy or incomplete biometric data. Recent research on biometric fusion has focused on developing new and improved feature fusion algorithms. As presented in [7], the authors proposed a feature-level Multi-biometric fusion algorithm called Dis-Eigen. They evaluated this algorithm on a dataset of face and fingerprint images and achieved an identification rate of 93.70%. In ref. [8], authors proposed a biometric recognition system using two feature extraction algorithms: the nearest neighbor algorithm for fingerprints and the speedup robust feature (SURF) algorithm for irises. Their system achieved an accuracy of 98.326%. As presented in [2], the authors evaluated a proposed model using deep features extracted from three popular pre-trained CNN models: AlexNet, VGG16, and GoogleNet. The model was tested on two benchmark datasets and achieved an accuracy of 99.05%. As presented in [9], the authors propose using fingerprint and palm print identification, two popular biometric systems, to improve the accuracy of individual identification. The authors use a deep neural network (DNN) to extract features from the fingerprint and palm print images and achieve an accuracy of 97.6%. As presented in [10], the authors propose a Multi-biometric identification system that combines fingerprint, palm print, and hand vein features. The system extracts features from each modality and combines them into a single feature vector. This feature vector is then used to generate a fuzzy vault, a secure way to store biometric data. In the recognition stage, the test person’s combined feature vector is compared to the fuzzy vault database to identify the individual. The proposed system was evaluated and achieved an accuracy of 98.5%. As presented in [11], the authors developed a face recognition system that uses the Rectangle Histogram of Oriented Gradients (R-HOG) feature extraction algorithm. The system achieves a low equal error rate (EER) and a high recognition accuracy of 99%. As presented in [12], the authors used different feature extraction algorithms for biometric modalities: 2-dimensional Principal Component Analysis (2DPCA) for the iris, Scale Invariant Feature Transform (SIFT) for signature, and Mel-frequency cepstral coefficients for speech. They then used a Genetic Algorithm to optimize the extracted features. Finally, they used an Artificial Neural Network (ANN) to classify the features and identify individuals. The proposed algorithm achieved an overall classification accuracy of 96–98%. Table 1 summarizes the recent techniques for feature fusion that have been noticed for their significant contributions to the multi-biometric authentication system.

### 2.2. Score-Level

Matching score level fusion is often used in applications where high accuracy is required [13], the authors proposed a model that first uses Principal Component Analysis (PCA) to extract features from 3D face images and then uses Iterative Closest Point (ICP) to extract features from 3D ear images. Finally, the model fuses the Face and ear features using score-level fusion. The proposed model achieves an accuracy of 99.25%. As presented in [3], the authors proposed a multimodal biometric framework that uses finger-knuckle-print (FKP) and iris features to authenticate individuals. It uses the scale-invariant feature transform (SIFT) and speeded-up robust features (SURF) algorithms to extract features from the FKP images and the Log Gabor wavelet to extract features from the iris images. The extracted features are then reduced in dimensionality using principal component analysis (PCA). The FKP and iris features are combined at the match score level using a neuro-fuzzy neural network classifier. The proposed framework was evaluated on the Poly-U and CASIA databases and achieved a promising recognition accuracy.

As presented in [14], the authors proposed a multimodal biometric person recognition system developed using a Convolutional Neural Network (CNN) for facial features and the Oriented FAST and Rotated BRIEF (ORB) algorithm for fingerprint features. The two features are fused at the match score level using a weighted sum rule, which achieves a recognition rate of 99.38%. As presented in [15], the authors proposed an improved local binary pattern (LBP) coding method to extract more robust face features. They also improve the conventional endpoint detection technology, voice activity detection (VAD), to more accurately detect voice mute and transition information, which boosts the effectiveness of voice matching. The proposed system achieves an accuracy of 98%.

As presented in [16], the authors proposed a way to combine the scores from multiple biometric systems, called weighted quasi-arithmetic mean (WQAM). WQAMs are estimated using different trigonometric functions. The proposed fusion scheme has the properties of both weighted mean and quasi-arithmetic mean and achieved a recognition rate of 97.22%. As presented in [17], the authors proposed an algorithm that preprocesses the palm print and face images and then extracts features from each image. The matching score for each trait is then calculated using the correlation coefficient. Finally, the matching scores are combined using t-norm-based score level fusion. The proposed algorithm achieves a Genuine Acceptance Rate (GAR) of 99.7%. As presented in [18], the authors used k-means clustering to divide the score range for each biometric modality into three zones of interest. They then apply two fusion approaches to the extracted regions: (1) decision tree combined with weighted sum (BCC) and (2) fuzzy logic (BFL). The BCC fusion approach achieves an accuracy of 95%, and the BFL fusion approach achieves an accuracy of 94.44%. Table 2 summarizes the recent techniques for score fusion that have been noticed for their significant contributions to Multi-biometric authentication systems.

Adaptive score fusion has recently made use of deep learning architectures. For example, Wang et al. [19] achieved state-of-the-art results on multimodal biometric benchmarks by proposing a gated attention network to dynamically learn modality-specific weights. Comparably, Zhang et al. [20] reported promising EER on a private dataset using a transformer-based fusion module that captures cross-modal dependencies at the score level. Although these techniques show remarkable accuracy, they frequently necessitate large labeled datasets, high processing power, and opaque weight assignment. The proposed SCA-based method, on the other hand, provides a lightweight, comprehensible, and data-efficient substitute that is especially well-suited for applications with limited resources or privacy concerns.

### 2.3. Decision-Level Fusion

Decision-level fusion is often used in applications with multiple biometric modalities, and/or the system must handle noisy or incomplete data. In ref. [4], authors proposed three decision-level fusion schemes for image recognition: Local Decision Fusion (LDF), Global Decision Fusion (GDF), and Local-Global Decision Fusion (LGDF). Their proposed LGDF method outperformed the feature-score hybrid fusion method by improving the average recognition rate by 6.75%. In ref. [21], the authors proposed a deep learning-based convolutional neural network architecture for classifying gender from fingerprints of each of the five finger types. They evaluate the performance of the proposed architecture and show that it improves classification accuracy by 18.72% overall, compared to the average classification accuracy of single classification models. In ref. [22], authors proposed a method for encrypting and compressing biometric data, which makes biometric data more secure and efficient for transmission over wireless networks. Better recognition rates are obtained when individual similarity scores are combined for the final decision. In ref. [23], authors presented a method to combine face and iris data for biometric systems, focusing on decision-level fusion to create a robust system. In ref. [24], authors proposed a fingerprint-based Multi-biometric cryptosystem (MBC) that uses decision-level fusion to improve security and accuracy. They also use hash functions to protect each biometric trait further. Experimental results and security analysis show that MBC outperforms single-biometric cryptosystems (SBCs) in security and accuracy. In ref. [25], authors proposed a fusion strategy that combines three classifiers based on feature and score-level fusion using a decision-level fusion rule. The strategy achieved a recognition accuracy of 98.75%. Table 3 summarizes the recent techniques for decision fusion that have been noticed for their significant contributions to Multi-biometric authentication systems.

## 3. Methodology

**Figure 1:**
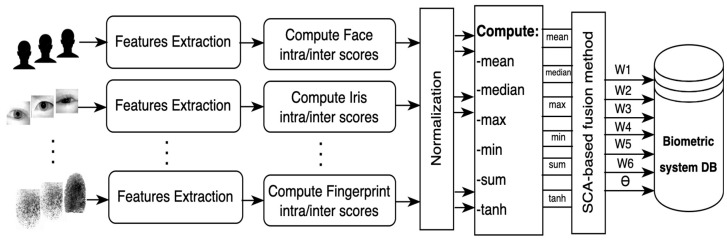
System Architecture of the Proposed SCA-based Multimodal Fusion Method.

Score fusion is the process of combining the matching scores from multiple biometric modalities to produce a single score representing the overall confidence that an individual is who they claim to be. Score fusion can improve the accuracy and robustness of biometric authentication systems by reducing the impact of noise and outliers from individual modalities. The proposed methodology utilizes SCA to find the optimal parameters for score fusion. Figure 1 shows the architecture diagram for the proposed fusion method. The system described in the figure can be used for various biometric authentication applications.

The system first extracts features from multiple biometric sources. The feature extraction step extracts the most relevant features from each biometric modality. These features are used to represent the biometric template of an individual. For the Face, these features may include facial landmarks, skin texture, and eye color. For the iris, the features may include the iris pattern. For the Fingerprint, the features may include the fingerprint pattern and minutiae. Once the features have been extracted, the system computes intra/inters scores for each modality. The intra-class scores for a given user using any biometric modality are the scores that measure the similarity between the user’s biometric templates. The inter-class scores for a given user using any biometric modality are the scores that measure the similarity between the user’s biometric templates and the biometric templates of other users.

A normalization process is needed since scores are computed using different biometric modalities. Score normalization is a process that converts the comparator’s parameters and data types into a common format. The three most used score normalization techniques are Min-Max, Z-Score, and Hyperbolic Tangent [26]. Then, the mean, maximum, minimum, median, summation, and Tanh are used to aggregate the scores for a given user using different biometric modalities. These aggregated scores can then be used to decide whether to accept or reject the user’s identity claim. The system uses an SCA-based fusion method to find the optimal parameters to fuse the normalized scores. The proposed SCA-based fusion method is a weighted sum method that assigns different weights to the scores from each modality based on their reliability using a training dataset of labeled biometric samples. It combines the normalized scores from different modalities into a single score.

Let K be the number of users in the Multi-biometric system, m be the number of biometric templates per user, and n be the size of each biometric template. Let $x_{i}^{r}$ be the $i^{th}$ biometric template for the kth user using the $r^{th}$ biometric modality. The intra-class scores for the kth user using the $r^{th}$ biometric modality is defined as follows (Equation (1)):

(1) $$X_{r}^{k}=\overset{m}{\underset{i=1}{⋃}}\overset{m}{\underset{j=i+1}{⋃}}d\left(x_{i},x_{j}\right)$$

where d(x, y) is the Euclidean distance between the templates, the inter-class scores for the kth user using the $r^{th}$ biometric modality is defined as follows (Equation (2)):

(2) $$Y_{r}^{k}={⋃}_{i=1}^{m}{⋃}_{j=1,j\neq i}^{K}d\left(x_{i},y_{j}\right)$$

$x_{i}$ is the ith biometric template for the ith user, and $y_{j}$ is the $j^{th}$ biometric template for the $j^{th}$ user. The previous computations are repeated for all available biometric modalities in the Multi-biometric system to compute $X_{r}$ and $Y_{r}$.

Given a total of R biometric modalities, the mean, maximum, minimum, median, summation, and Tanh intra scores are defined, respectively, as follows (Equations (3)–(8)):

(3) $$X_{mean}=mean(X_{1},...{,X}_{r},...,X_{R})$$

(4) $$X_{max}=max(X_{1},...{,X}_{r},...,X_{R})$$

(5) $$X_{min}=min(X_{1},...{,X}_{r},...,X_{R})$$

(6) $$X_{median}=median(X_{1},...,X_{r},...,X_{R})$$

(7) $$X_{sum}=X_{1}+...+X_{r}+...+X_{R}$$

(8) $$X_{tanh}=Tanh(X_{1},...,X_{r},...,X_{R})$$

The same definitions can compute the mean, maximum, minimum, median, summation, and Tanh inter scores $Y_{mean}$, $\;Y_{max}$, $\;Y_{min}$, $Y_{median}$, $Y_{sum}$, $\;Y_{tanh}$.

The SCA-based fusion algorithm must first be initialized with a population of random solutions. Each solution represents a set of parameters for the matching scores from the different biometric modalities. The SCA then iteratively updates the population of solutions until it finds a solution that produces the lowest equal error rate. The candidate solution is represented by a vector I, where I = {$w_{1}$, $w_{2}$, $w_{3}$, $w_{4}$, $w_{5}$, $w_{6}$, θ}. Where θ ∊ [lb, ub], and the weights $w_{i}$ are in the range [0, 1] and subject to the constraint in (Equation (9)):

(9) $$\sum_{i=1}^{6}w_{i}=1$$

The weight values are used to compute the fused intra/inter scores for the training set, respectively, as follows (Equations (10) and (11)):

(10) $$X_{fused}=w_{1}{\cdot X}_{mean}+w_{2}{\cdot X}_{max}+{w_{3}\cdot X}_{min}+{w_{4}\cdot X}_{median}+{w_{5}\cdot X}_{sum}+w_{6}{\cdot X}_{tanh}$$

(11) $$Y_{fused}=w_{1}{\cdot Y}_{mean}+w_{2}{\cdot Y}_{max}+{w_{3}\cdot Y}_{min}+{w_{4}\cdot Y}_{median}+{w_{5}\cdot Y}_{sum}+w_{6}{\cdot Y}_{tanh}$$

During the evolution of the SCA population to find the best fusion parameters, it is necessary to ensure that the candidate solutions are bound to the search space, as follows (Equation (12)):

(12) $$I=\left\{\begin{array}{c} I_{new},\;\mathrm{if}\;(\sum_{i=1}^{6}w_{i}\neq 1)||(\sum_{i=1}^{6}{(w}_{i}>1)\neq 0)||(\sum_{i=1}^{6}{(w}_{i}<0)\neq 0)\\ I,\;\mathrm{otherwise} \end{array}\right.$$

Each candidate solution is evaluated using its fitness value f, representing the biometric system’s equal error rate (EER). The target is to minimize the error rate. The fused scores are used to compute the EER for the biometric system based on the given threshold. The fitness value, f, for each candidate solution, is computed as follows (Equations (13)–(15)):

(13) $$FAR=\frac{\sum_{i=1}^{\left|Y_{fused}\right|}\left(Y_{fused}^{i}<\theta \right)}{\left|Y_{fused}\right|}\times 100$$

(14) $$FRR=\frac{\sum_{i=1}^{\left|X_{fused}\right|}\left(X_{fused}^{i}\geq \theta \right)}{\left|X_{fused}\right|}\times 100$$

(15) $$f=\frac{\left(FAR+FRR\right)}{2}$$

The pseudo code of the proposed SCA-based score fusion algorithm is given by Algorithm 1.
**Algorithm 1.** SCA-based score fusion algorithm**Input:** Population size  (p), number of iterations $\;(t_{max})$, the mean intra/inter scores ($X_{mean}\;/\;Y_{mean}$), the maximum intra/inter scores ($X_{max}\;/\;Y_{max}$), the minimum intra/inter scores ($X_{min}\;/\;Y_{min}$), the median intra/inter scores ($X_{median}\;/\;Y_{median}$), the sum intra/inter scores ($X_{sum}\;/\;Y_{sum}$), and tanh intra/inter scores ($X_{tanh}\;/\;Y_{tanh}$). **Output:** The best score fusion parameters $I_{best}$.
**1**    Create initial population ${\;Pop}_{0}$ that includes p candidate solutions I each of length 7.**2**    Calculate the fitness value for each solution I in the population ${\;Pop}_{0}$ using Equation (15).**3**    Obtain the best/minimum fitness value $f_{best}$ in the population ${\;Pop}_{0}$.**4**    Achieve the corresponding best solution $I_{best}$ in the population ${\;Pop}_{0}$.**5**    Set the initial values of $r_{1}$ and ${\;r}_{2}$.**6**       **For** t = 2 to $t_{max}$**7**    Set the tuning parameter a  to 2.**8**       Set $r_{1}$ = a − t × ((a)/$t_{max}$).**9**             **For** i = 1 to p**10**               **For** j = 1 to 7**11**         Set $r_{2}$ = (2 × π) × r.                  /*r ∊ ℝ, r ∊ [0, 1] */    Set $r_{3}\;$= 2*r.**12**        Set $r_{4}$ = r.**13**       **If** $r_{4}<0.5$ **Then,****14**       I(i,j)=I(i,j)+($r_{1}\;\times$ sin($r_{2}$) × abs($r_{3}\times I_{best}$(j) − I(i, j))).**15**        **Else****16**       I(i,j)=I(i,j)+($r_{1}\;\times$ cos($r_{2}$) × abs($r_{3}\times I_{best}$(j) − I(i, j))).**17**       **End****18**       **End****19**       **End****20**       Ensure that each solution is in the population within the search space [Equation (12)].**21**       Calculate the fitness value for each solution I in the population ${Pop}_{t}$ using Equation (15).**22**       Update the best fitness value $f_{best}$ in the population ${\;Pop}_{t}$.**23**       Update the corresponding best solution $I_{best}$ in the population ${\;Pop}_{t}$.**24**    **End****25**    Return the best-found solution ($I_{best}$) in the last population.


The suggested fusion framework is modality-independent, meaning that any biometric modality (such as voice, palm print, or fingerprint) that generates a scalar match score is accepted. The score normalization step allows for this generality by mapping different score ranges into a common [0, 1] interval. Plug-and-play deployment is made possible by the fact that the same SCA-based optimization pipeline can be applied to any combination of modalities without requiring architectural modifications. The implementation details and the performance evaluation of the proposed algorithm are explained in detail in the next section.

## 4. Result Evaluation and Discussion

This section explains how our proposed method significantly improves recognition performance compared to unimodal biometric systems and highlights the most important contribution. We conducted experiments using the CASIA iris dataset [27] and the AT&T (ORL) face database [28] to evaluate the proposed method. The CASIA-V3-Internal iris dataset contains 146 subjects, each with images of their left and right eyes. We preprocessed the images by segmenting, normalizing, and converting them to binary iris codes using the Libron Mask code [29]. The Libron Mask code is a multi-step process that includes iris segmentation and localization using the Circular Hough Transform and Linear Hough Transform, followed by normalization using Daugman’s rubber sheet model, and finally, encoding the normalized iris region using 1D Log-Gabor filters and phase quantization to create binary iris templates. We then reshaped each iris template into a single binary iris code vector, which we used as input for the experiments. The AT&T (ORL) face database comprises 400 images of 40 individuals, each represented by ten facial photos exhibiting variations in facial expressions, lighting, and time. These images were captured against a plain black background and are 92 × 112 pixels in size, with 256 gray levels per pixel. Binary facial features were extracted using an optimized Genetic algorithm transformation [30] applied to the features extracted using principal component analysis (PCA) [31]. In this experiment, 40 subjects were randomly selected from the CASIA-V3-Internal iris dataset and assigned to the ORL face dataset. During the experiments, left and right iris samples were paired with face samples for each of the 40 subjects. The samples were divided into two groups, with 60% allocated for training and 40% for testing. The generated scores are normalized by dividing by their length to account for the different lengths of binary iris and face templates. This ensures that all biometric scores are on the same scale. Multiple experiments were conducted using the gray wolf optimizer and Practical Swarm Optimizer to comprehensively evaluate the proposed method and compare it to other metaheuristic algorithms in the literature [32]. Experimental parameters are presented in Table 4, including fixed parameter values shared between the applied metaheuristic algorithms to facilitate equitable comparison of outcomes. Moreover, to account for the stochasticity of metaheuristic algorithms and generate robust results, we repeat each experiment ten times and report the mean results.

Four experiments are performed to compare the accuracy of the proposed fusion method with the original unimodal biometric. The proposed fusion method was evaluated on four levels: left iris with right iris, left iris with Face, right iris with Face, and left and right iris with Face. The following metrics are used to evaluate the recognition accuracy: False Accepted Rate (FAR), False Rejection Rate (FRR), and Equal Error Rate (EER). The False Acceptance Rate of an identification system is the percentage of times the system incorrectly grants access to an unauthorized person. The False Rejection Rate, on the other hand, is the percentage of times that the system incorrectly denies access to an authorized person. The Equal Error Rate is where the FAR and FRR are equal, meaning the system is equally likely to make either error [33]. Table 5 shows the results compared to the original unimodal system.

Based on the results in the table, the proposed fusion method performs well on all four levels, with an EER below 5% for all combinations of models. The lowest EER is achieved by fusing the left and right iris with the Face, which is 1.003%. This suggests that the proposed fusion method effectively combines scores from different models to improve the overall accuracy of the identification system. The explanation for the proposed fusion method’s good performance is that it considers the complementary nature of different features. For example, the iris is a unique identifier difficult to forge or alter. The Face, on the other hand, is more susceptible to appearance changes due to aging, lighting conditions, and facial expressions. However, the Face can also provide additional information about the individual, such as gender, ethnicity, and age. By fusing information from both the iris and the Face, the proposed method can achieve higher accuracy than could be achieved with either feature alone.

Utilizing anatomical symmetry, the left + right iris fusion produces limited modality diversity but high intra-class consistency. A spoof-resistant trait (iris) and a user-friendly trait (face) are combined in face + iris fusion; however, face performance deteriorates in low light or when expressions change. For high-security settings where hardware overhead is acceptable, the tri-modal fusion (left + right iris + face) maximizes complementary information and achieves the lowest EER (1.003%) at the expense of requiring sensors.

**Figure 2:**
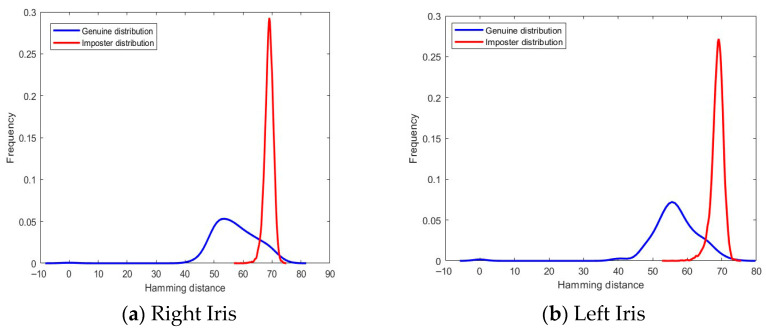

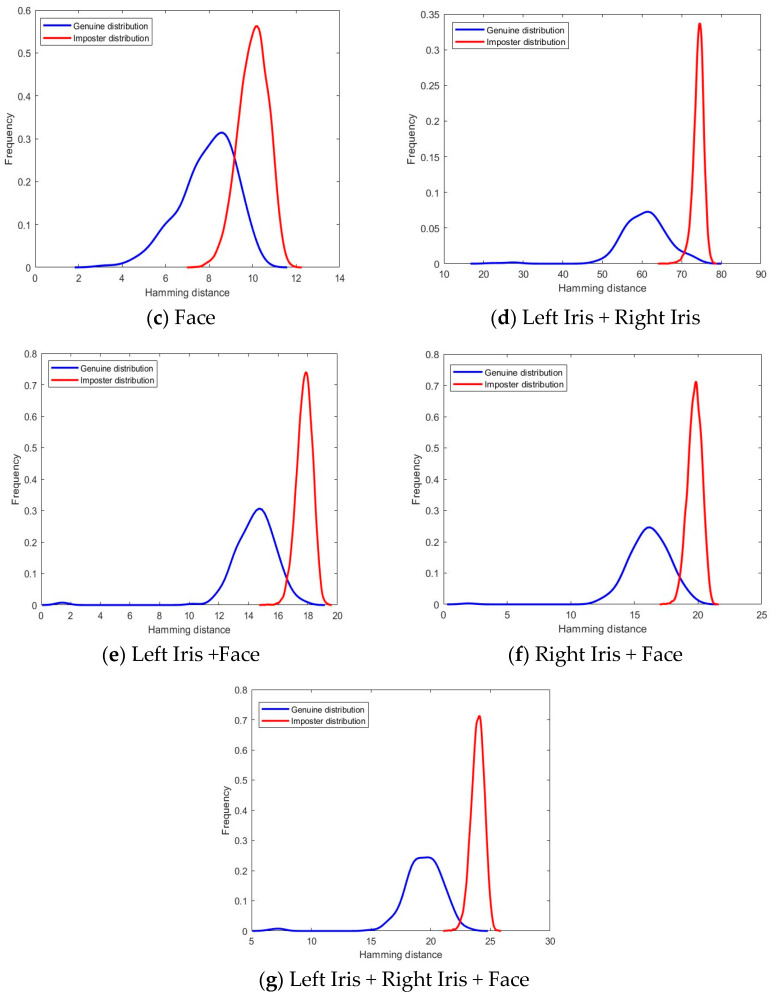
Genuine and imposter distributions for all experiments.

Moreover, the proposed SCA optimizer finds the optimal weight values for each score and the optimal threshold for the final decision. This is important because different scores may have different levels of importance, and different thresholds may be appropriate for the recognition accuracy. By using the SCA optimizer to set the fusion parameters, the proposed fusion method can achieve a higher level of accuracy than could be achieved with a fixed set of parameters. Overall, the results in the table suggest that the proposed fusion method is a promising approach for improving the accuracy of identification systems. Furthermore, the genuine and imposter distributions are computed by considering all feasible database comparisons using the Hamming distance measure. The genuine and imposter distributions are visualized in Figure 2.

**Figure 3:**
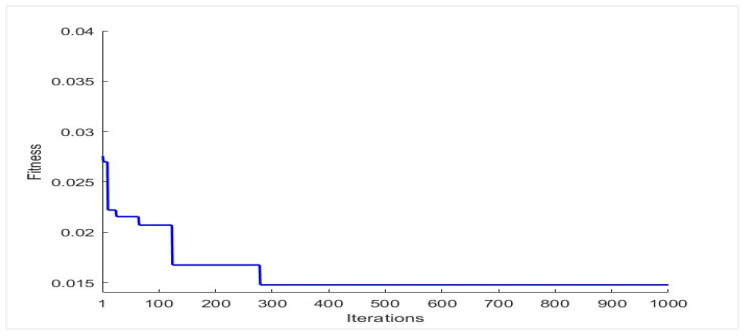
Convergence curve for the proposed fusion using SCA.

The figure shows that the mean values of the genuine distribution are 57.22, 56.38, 7.91, 60.56, 14.41, 16.12, and 19.33 for the unimodal right iris, unimodal left iris, unimodal Face, proposed fusion of left and right iris, proposed fusion of left iris and Face, proposed fusion of right iris and Face, and proposed fusion of left and right iris with Face, respectively. The mean values of the imposter distribution are 68.95, 68.74, 10.02, 74.21, 17.78, 19.74, and 23.94 for the same modalities, respectively. This indicates that the proposed score-level fusion scheme significantly improves the separation between the genuine and imposter distributions compared to unimodal biometric systems. To further analyze the proposed method, the convergence curve for the proposed SCA optimizer is depicted in Figure 3. The curve represents the experiment for the proposed fusion of the training data, which contains the left and right iris with the Face.

The convergence curve for the SCA shows that the algorithm converges to a good solution within a reasonable number of iterations and achieves a low EER. The curve indicates that the algorithm is progressing consistently towards the optimal solution. The good performance of the SCA is achieved through a sufficient number of candidate solutions and evolves over time. This allows the SCA to explore a large region of the solution space and find the optimal solution. Moreover, SCA uses sine and cosine operators, allowing the SCA to explore the solution space more efficiently than traditional search operators.

Despite the lack of explicit spoofing experiments, the suggested fusion framework shows intrinsic resilience to presentation and noise attacks. First, by combining complementary modalities (such as face and texture-rich iris), the system lessens dependence on any one vulnerable characteristic. Second, during fusion, compromised or noisy modalities are automatically downweighed by the SCA-optimized weights, as shown by the better separation of real and fake distributions (Figure 2). This behavior is consistent with research in [33], which demonstrated that score-level fusion improved resilience to partial occlusion and sensor noise. Future research will involve formal evaluation under spoofing protocols, such as CASIA-Iris-AntiSpoofing or LivDet.

Two additional experiments are conducted using the Gray Wolf Optimizer (GWO) and the Practical Swarm Optimizer (PSO) to comprehensively evaluate the proposed fusion method and compare it to other metaheuristic algorithms. For all applied experiments, Table 6, Table 7 and Table 8 show the EER, the decidability metrics, and the accuracy improvement ratio.

Table 6 shows that the SCA outperformed the GWO and PSO algorithms on all four models. SCA is more effective at finding the optimal parameters for the biometric systems. The superior performance of the SCA is that it is more effective at exploring the search space and finding the global optimum than search algorithms such as GWO and PSO. We employ Equal Error Rate (EER) as the main metric because of its threshold-invariance and cross-study comparability, in accordance with accepted practice in biometric evaluation [33]. To give a complete picture of system performance, we also report FAR, FRR (Table 5), and decidability index d’ (Table 7).

The results in Table 7 show that the SCA outperformed the GWO and PSO algorithms on all four biometric models regarding the decidability metrics. d’ denotes the decidability metrics that indicate the separation between the genuine and impostor distributions, which is defined by (16):

(16) $$d’=\frac{\left|\mu_{i}-\mu_{g}\right|}{\sqrt{\frac{\sigma_{i}^{2}+\sigma_{g}^{2}}{2}}}$$

where $\mu_{i}$ and are $\mu_{g}$ the means and ${\sigma i}^{2}$ and ${\sigma g}^{2}$ are the variances of the imposter and genuine distributions, respectively. Hence, the largest d’ value indicates higher recognition performance. SCA is more effective at discriminating between genuine and impostor users.

The results in Table 8 show that the SCA significantly outperformed the GWO and PSO algorithms on all four biometric models regarding the improvement ratio. The ratio is computed compared to the original unimodal system; it is defined by (Equation (17)):

(17) $$Improvement=\frac{min\left(\left(EER_{m_{1}},\ldots ,\;EER_{m_{n}}\right)\right)\left(-\left(EER_{m_{1}+\ldots +m_{n}}\right)\right)}{min\left(EER_{m_{1}},\ldots ,\;EER_{m_{n}}\right)}\times 100$$

where min (${EER}_{m_{1}},{...,EER}_{m_{n}}$) denotes the minimum EER among the unimodal m_1_ to m_2_, and ${EER}_{m_{1}+...+m_{n}}$ denotes the EER for the fused model using the biometric models m_1_ to m_2_. The table shows that the performance improvement ranged from 53.58% to 85.89%. This suggests that the SCA is much more effective for optimizing biometric systems than the GWO and PSO algorithms.

This experiment investigates the elapsed run time of the applied metaheuristic algorithms. For a fair comparison, the population size and iterations are fixed (as shown in Table 4), and all algorithms are implemented using MATLAB R2023a and performed on the same machine with Processor AMD Ryzen 7, CPU 3.20 GHz, 16 GB memory. The obtained results are shown in Table 9.

The results in Table 9 show that the SCA outperformed the GWO and PSO algorithms regarding running time. The SCA completed the task in 9410 milliseconds, while the GWO and PSO algorithms took 9800 and 10,940 milliseconds, respectively. Eventually, the proposed score fusion method is compared to other multimodal biometric methods in the literature. Table 10 shows the results of this comparison, including the type of biometric data, the fusion type, and EER.

It is noteworthy that the SCA optimization is not carried out during live authentication, but rather only once during system enrollment or periodic recalibration. A straightforward weighted sum of normalized scores is used to calculate the fused score at runtime, requiring very little latency (<1 ms per query). Thus, the 9410 ms total optimization time is a one-time offline expense that is acceptable even on edge devices for high-security systems. This design maintains adaptive weight learning while guaranteeing real-time responsiveness during authentication.

The proposed method of fusing the left and right iris with the Face achieved promising recognition performance compared to other methods. However, deep learning-based score fusion methods achieved better recognition accuracy on average, even though they required more computational resources. This can be a limiting factor for resource-constrained applications.

## 5. Conclusions

This paper proposes a new score fusion method based on the Sine Cosine Algorithm (SCA). The proposed method extracts features from multiple biometric sources and then computes intra/inter scores for each modality. The proposed method then normalizes the scores for a given user using different biometric modalities and aggregates them using various aggregation rules. The role of the SCA is to find the optimal parameters to fuse the normalized scores. We evaluated the proposed method on the CASIA-V3-Internal iris dataset and the AT&T (ORL) face database. The results showed that the improvement in the recognition accuracy ranged from 53.58% to 85.89% compared to the unimodal biometric systems. Moreover, it outperforms several state-of-the-art score fusion methods regarding accuracy and robustness. Furthermore, the proposed method learns to fuse the scores from different modalities based on a training dataset of labeled biometric samples. This allows the algorithm to adapt to the biometric modalities’ specific characteristics. Moreover, the proposed method is relatively easy to implement and can be used with various biometric modalities. This work has direct applicability in high-assurance identity verification scenarios. For example, border control can integrate iris and face to balance security and user throughput; mobile banking apps can use face with behavioral biometrics for frictionless yet spoof-resistant login; and telehealth platforms can prevent patient misidentification in remote consultations. The method’s low runtime overhead and modality flexibility make it suitable for both cloud and edge deployments. Future research will concentrate on assessing robustness under standardized spoofing benchmarks and putting the fusion pipeline on edge hardware to evaluate real-world latency and power consumption, and investigating hybrid fusion strategies that combine SCA with lightweight neural post-processors to capture nonlinear score interactions while maintaining interpretability.

## Figures and Tables

**Table 1:** State-of-the-Art Feature-Level Fusion Techniques, NM: actual dataset size used in the experiment is not mention in the study.

Biometric Trait Used	Algorithm	Database	Size	Recognition Accuracy
Face & Fingerprint	Dis-Eigen [7]	AUMI	160	93.7%
Iris& Fingerprint	ANN Trained and WOA optimized [8]	CASIA V1.0 & FVC2004 DB1	100	98.3%
Ear & profile face	CNN (Alex Net, VGG16 and Google Net) [2]	UND-E & UND-j2	114; 273	99%
Fingerprint & Palmprint	DNN [9]	Chimeric	NM	97.6%
Fingerprint, Palmprint &Hand-vein	Fuzzy Vault [10]	CASIA	NM	98.5%
Iris & Face	R-HoG [11]	SDUMLA-HMT	NM	99%
Iris, Speech & Signature	2D PCA, SIFT, ANN Classifier [12]	A mixture of standard and real-time data set	500	96–98%

**Table 2:** State-of-the-Art Score-Level Fusion Techniques, NM: ‘actual dataset size used in the experiment is not mentioned in the study’.

Biometric Trait Used	Algorithm	Database	Size	Recognition Accuracy
3D-face & 3D-ear	PCA for 3D face & ICP for 3D ear [13]	FRGC & UND-F, G	557; 302; 235	99.25%
Iris & finger-knuckle-print	SIFT, PCA, neuro-fuzzy neural network [3]	CASIA & Poly-U	NM	98%
Face & Fingerprint	CNN & ORB [14]	UCI Database	400; 120	99.38%
Face & Voice	LBP & VAD [15]	XJTU Database	102	98%
Fingerprint & Face	WQAM [16]	NIST-BSSR1	517	97.22%
Palm print & Face	ROI- t-norm [17]	Face 94, Face 95, Face 96, FERET, FRGC & IITD	600	99.7%
Iris & Fingerprint	BCC, BFL, K-means, Decision Tree and Fuzzy Logic [18]	CASIA Iris V4 & CASIA Fingerprint V5	1100	94.4–95%

**Table 3:** State-of-the-Art Decision-Level Fusion Techniques.

Biometric Trait Used	Algorithm	Database	Size	Recognition Accuracy
Palmprint & Face	Wavelet sub-bands, Nearest Neighbor Classifier [4]	ORL & Yale & ILT-Delhi	330	98.12%
Multiple Fingerprint	CNN [21]	Novel dataset	500	94.7%
Face & fingerprint	Joint Encryption and Compression technique [22]	FEI & NIST	400	97%
Face & Iris	OR rule [23]	CASIA-Iris-Distance	1420	98.9%
Fingerprints of different fingers	Multi-finger feature encrypted by a hash function [24].	Novel dataset	1500	95.76%
Face & Iris	Majority voting [25]	ORL & CASIA	400	98.75%

**Table 4:** Experimental parameters.

Parameters	Value	Algorithm
Pop size	150	SCA + PSO + GWO
Iterations	1000	
Solution dimension	7	
a	2.0	SCA + GWO
Inertia weight(w)	1.0	PSO
Acceleration coefficients (c1)	2.0	PSO
Acceleration coefficients (c2)	2.0	PSO

**Table 5:** Performance results.

Model	FAR (%)	FRR (%)	EER (%)
Left Iris	1.64	12.58	7.11
Right Iris	8.54	15.00	11.77
Face	4.61	21.17	12.89
Proposed (Left iris + Right iris)	1.48	5.13	3.30
Proposed (Left iris + Face)	1.33	4.50	2.91
Proposed (Right iris + Face)	3.79	5.29	4.54
Proposed (Left iris + Right iris + Face)	0.76	1.25	1.003

**Table 6:** EER (%) for the applied metaheuristic algorithms.

Model	SCA	GWO	PSO
Left iris + Right iris	3.30	5.74	3.31
Left iris + Face	2.91	4.38	3.42
Right iris + Face	4.54	6.55	4.53
Left iris + Right iris + Face	1.003	3.41	1.49

**Table 7:** d’ for the applied metaheuristic algorithms.

Model	SCA	GWO	PSO
Left iris + Right iris	6.918	5.560	4.972
Left iris + Face	3.606	2.954	3.575
Right iris + Face	3.326	3.435	3.343
Left iris + Right iris + Face	5.683	4.429	5.582

**Table 8:** Improvement ratio (%) for the applied metaheuristic algorithms.

Model	SCA	GWO	PSO
Left iris +Right iris	53.58	19.27	53.45
Left iris + Face	59.07	38.40	51.90
Right iris + Face	61.43	44.35	61.51
Left iris + Right iris + Face	85.89	52.03	79.04

**Table 9:** Running time (in milliseconds) for the applied metaheuristic algorithms.

Method	Time (ms)
SCA	9410
GWO	9800
PSO	10,940

**Table 10:** Comparative study analysis.

Reference	Year	Biometric Modality	Fusion Type	EER (%)
[12]	2020	Iris + Signature + Speech	Feature-Level	4.00
[25]	2015	Face + Iris	Decision-Level	1.25
[14]	2021	Face + Fingerprints	Score-Level	0.62
[16]	2020	Face + Fingerprints	Score-Level	2.78
Proposed	-	Left iris + Right iris	Score-Level	3.30
Proposed	-	Left iris + Face	Score-Level	2.91
Proposed	-	Right iris + Face	Score-Level	4.54
Proposed	-	Left iris + Right iris + Face	Score-Level	1.00

## Data Availability

The used datasets are publicly available on: http://cam-orl.co.uk/facedatabase.html, accessed on 24 December 2025 [AT&T (ORL) face data set link]; http://biometrics.idealtest.org, accessed on 24 December 2025 [CASIA-V3-Internal iris data set link]; Implementation code used for the current study is provided upon reasonable requests to authors.
